# Cross Tissue Trait-Pathway Network Reveals the Importance of Oxidative Stress and Inflammation Pathways in Obesity-Induced Diabetes in Mouse

**DOI:** 10.1371/journal.pone.0044544

**Published:** 2012-09-17

**Authors:** Shouguo Gao, Herbert Keith Roberts, Xujing Wang

**Affiliations:** 1 Department of Physics, University of Alabama at Birmingham, Birmingham, Alabama, United States of America; 2 The Comprehensive Diabetes Center, University of Alabama at Birmingham, Birmingham, Alabama, United States of America; Centro Cardiologico Monzino IRCCS, Italy

## Abstract

Complex disorders often involve dysfunctions in multiple tissue organs. Elucidating the communication among them is important to understanding disease pathophysiology. In this study we integrate multiple tissue gene expression and quantitative trait measurements of an obesity-induced diabetes mouse model, with databases of molecular interaction networks, to construct a cross tissue trait-pathway network. The animals belong to two strains of mice (BTBR or B6), of two obesity status (obese or lean), and at two different ages (4 weeks and 10 weeks). Only 10 week obese BTBR animals are diabetic. The expression data was first utilized to determine the state of every pathway in each tissue, which is subsequently utilized to construct a pathway co-expression network and to define trait-relevant and trait-linking pathways. Among the six tissues profiled, the adipose contains the largest number of trait-linking pathways. Among the eight traits measured, the body weight and plasma insulin level possess the most number of relevant and linking pathways. Topological analysis of the trait-pathway network revealed that the glycolysis/gluconeogenesis pathway in liver and the insulin signaling pathway in muscle are of top importance to the information flow in the network, with the highest degrees and betweenness centralities. Interestingly, pathways related to metabolism and oxidative stress actively interact with many other pathways in all animals, whereas, among the 10 week animals, the inflammation pathways were preferentially interactive in the diabetic ones only. In summary, our method offers a systems approach to delineate disease trait relevant intra- and cross tissue pathway interactions, and provides insights to the molecular basis of the obesity-induced diabetes.

## Introduction

Phenotypic traits are properties that emerge from the interactions of genes within a dynamic environmental framework. A major goal of systems biology is to understand how the interactions lead to the observed traits [1], [2]. This is especially critical in the study of complex diseases, where it is evident that they cannot be deciphered through considering individual genes only. A disease trait normally correlates with the inability of a particular functional network module to carry out its basic function, and the pathogenesis of a complex disease can involve the perturbations of more than one module. In a complex disease like Type 2 Diabetes (T2D), a spectrum of traits, such as obesity, hyperglycemia, insulin resistance, etc, are associated to the risk of developing T2D. These suggest that multiple functional pathways are involved. Further complexities arise from the fact that multiple tissues are involved and the crosstalk among them is important to disease development. For instance, problems in both the insulin secreting pancreatic islets and target tissues of insulin action are observed in diabetics, and are believed to contribute to disease pathogenesis [3]–[8]. Therefore it will be highly valuable to map out the signaling pathways, and their interactions, both intra and cross tissue, underlying the disease traits.

A basic bioinformatics question that arises is, in mapping the genetic architecture of a disease, is it more efficient to develop gene level metrics and make assessment of gene networks through their members' relevance to disease; or to develop network level metrics that directly assess a whole network? In the gene expression data analysis, both approaches were developed, either starting with differentially expressed genes followed by identifying pathways with enhanced presentation among them; or starting with pathways (or predefined gene set) directly through evaluating the expression distribution shift of the whole gene set [9].

Networking individual genes is known to suffer from high noise and high false positive rate. On the other hand, networking pathways has demonstrated its advantage in providing more relevant biological insights in understanding disease pathogenesis and in establishing the inter- disease relationships [3], [10]. For instance, Hu and Li proposed a framework to construct a network of pathways according to co-expression between genes in different pathways [3]. Pathways relevant to each disease are ascertained from the disease induced differential expression of their members. When applied to T2D and obesity, they demonstrated that the method can identify signature pathways for each disease and establish valid association between them. Li *et al* proposed an approach that first defines disease associated genes through literature mining, followed by associating pathways to diseases based on enriched disease gene presence among pathway members, and linking different diseases based on pathway sharing [11]. To our knowledge, there is no study till now that focuses on delineating the association of clinical traits related to the same disease at the molecular pathway level.

Recently, Keller et al profiled gene expression in six tissues (pancreatic islet, liver, adipose, hypothalamus, gastrocnemius, and soleus), and measured eight quantitative traits (including plasma, glucose, and insulin) in a mouse model of obesity-induced diabetes [7]. They constructed co-expression networks in each tissue and linked network modules (densely connected subregions of the whole network) to traits if the average expression of members correlates to trait variations. A number of modules in islet, adipose, and soleus were found to strongly correlate with plasma glucose; several modules in islets, liver, and adipose exhibited a high correlation with insulin but not with glucose; a module in adipose correlated with inflammation. They further constructed intra- and inter-tissue networks of the pathways by linking those whose first principal components correlated. It was found that there was substantial tissue difference in the degree of intra-tissue connectivity. In both BTBR and B6 mouse strains, the two muscle tissues had the most intra-tissue connections (i.e. co-expressions), whereas liver and hypothalamus had the fewest. There was also significant strain difference, likely obesity-dependent, in the structure of the intra-tissue co-expression network. For instance, profiles of the cell cycle module suggested that obesity induced islet cellular proliferation occurred in B6 but not in BTBR mice. There was also an increase in inter-tissue connectivity in BTBR vs B6.

In a similar study by Dobrin et al [8], gene expression levels were profiled in adipose, liver and hypothalamus of the F2 progeny from a cross between the outbred M16 and ICR (imprinting Control Region) control mouse strains. The bipartite tissue to tissue co-expression network was first constructed for each pair of tissues, and subsequently partitioned into subnetworks utilizing the edge betweenness centrality measures defined in [12]. Centrality of a network node refers to its relative importance in the network, in terms of its potential influence on others. Edge betweenness is one of the centrality measures that was first proposed by Linton Freeman [12], which focuses on evaluating a node's importance both to the local structure and to the global information flow. It is defined to be the fraction of shortest paths between all other nodes that passes through the given node. Topological evaluation of the network revealed that it is scale free, with some genes acting as hub nodes operating across tissues. Through analysis of the cross tissue networks, entire new classes of genes were identified to be associated with disease [8]. These genes were systematically overlooked in single tissue analyses because they formed, on average, no meaningful intra-tissue connections.

In both studies [7], [8], pathway networks were constructed using individual gene-based metrics; the disease trait measurements were not utilized. Here we propose a pipeline to reverse engineer the tissue-specific pathway and trait interaction networks for obesity-induced T2D using the gene expression and trait measurements by Keller et al [7]. We will utilize the Pathway Connectivity Index (PCI) that we previously developed to characterize the molecular state of a pathway during a given biological process [13], and to link the different pathways, and to link pathways to traits. PCI defines the molecular state of a network based both on the state of individual genes and on the topological structure of their interactions (see Methods). We have previously demonstrated its efficiency at identifying phenotype associated pathways using several gene expression datasets [13]–[15] and in candidate disease gene prediction [16], [17]. Pathway network edges are defined if the PCI of two pathways correlate, and pathway-trait links are made if the pathway PCI correlates to trait variations. By performing comparative network analysis, we can then hypothesize about the association among clinical traits at the molecular pathway level.

Compared to the existing works [7], [8], our approach is new and different in the following aspect: (1) We start with the known KEGG pathways (www.genome.jp/kegg/) as the unit of our operation. We directly characterize the state of each pathway using a new quantitative metric that considers contributions both from individual genes and from their interactions. (2) The approach by Keller et al restricted their analysis to genes that showed one of the 15 predefined patterns [7], and the study by Dobrin et al [8] restricted to genes showing differential expression in at least 5% of the samples. Our approach utilizes expression information of every gene to score pathways and therefore will not miss gene sets that exhibit subtle but consistent changes as a group [9]. (3) Though eight disease related traits were measured in every animal by Keller et al [7], they were not previously utilized in pathway network construction nor in identification of disease-relevant pathways. We will include them to delineate the genetic architecture underlying each trait.

## Materials and Methods

### Gene expression and quantitative trait data

Multi-tissue microarray data were obtained from Gene Expression Omnibus (GEO, http://www.ncbi.nlm.nih.gov/geo/, GSE10785). In this study [7], gene expression levels were profiled in six tissues that include hypothalamus, gastrocnemius, soleus, liver, adipose, and pancreatic islet from 2 mouse strains, the lean and obese C57BL/6 (B6) and the lean and obese BTBR mice, at two different ages, 4 weeks and 10 weeks. Five replicate animals were sacrificed under each condition in each strain, totaling 40 mice (2 strains×2 obesity status×2 time points×5 replicate animals). The two strains differ in obesity-induced diabetes susceptibility. B6 mice remain essentially non-diabetic at all ages, irrespective of obesity; whilst obese BTBR mice become severely diabetic by 10 wk of age. This study used Rosetta/Merck Mouse 44 k 1.0 oligonucleotide microarray. For a given tissue from each animal, labeled cRNA was hybridized against a pool sample constructed from equal aliquots of RNA from all of the 20 animals of the corresponding strain. This gives a total of 240 microarrays (6 tissues×40 animals). In our study we used the log10 ratio (of each animal to the common reference) in our network modeling.

Eight T2D-related quantitative traits were also measured in each mouse and kindly provided to us by the authors [7]. These include the plasma glucose, insulin, total number of islets harvested per pancreas, body weight, triglyceride (TG), and levels in circulation of three adipokines: adiponectin, plasminogen activator inhibitor-1 (PAI-1), and resistin.

### Mouse pathway and gene interaction network information

187 mouse pathways were downloaded from KEGG (http://www.genome.jp/kegg), and a Perl script is written to curate the information of pathway links maintained in KEGG. Functional interactions of mouse genes were downloaded from the Princeton mouseNET (http://avis.princeton.edu/mouseNET/). mouseNET uses probabilistic framework to integrate diverse genetic and genomic data to generate a functional network for the laboratory mouse. It allows the users to predict novel functional assignments and network components [18].

### Characterizing the activity state of a pathway

To network pathways one needs to have a quantitative metric to characterize the state of each pathway. We believe that an efficient metric needs to capture not only the activities of individual genes in the pathway, but also their interaction patterns. Given a pathway of *n* genes 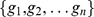, its gene interaction network can be represented by a graph 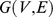, where vertex set 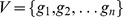, and the edge set 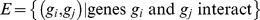; or a matrix called the adjacency matrix 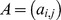, where 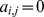, if genes *i* and *j* do not interact; and 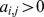 otherwise. In this study we construct *E* based on the mouseNET, and the adjacency matrix is defined as:
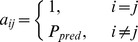
(1)where 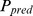 is the probability of functional linkage between the two genes predicted by the mouseNET [18].

Assuming that 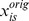 is the (original) log expression measurement for gene *i* in sample *s*, we first normalize it to zero mean and unit variance through 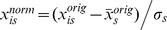, and then transform it using the Sigmoid function 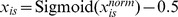. For each pathway we subsequently define the Pathway Connectivity Index, PCI, to capture pathway level activity [13]:
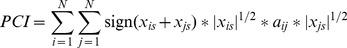
(2)where *N* is the number of genes in the pathway. This concept was first introduced in our previous studies [13], and the adjacency matrix of the protein-protein interaction network of genes in a pathway was used in the definition. 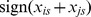 represents the overall expression status (up- or down-regulation) of the gene pair, and helps to reduce the information loss resulted from using absolute expression values. The Sigmoid transformation is introduced to reduce impact to PCI by genes with extremely low or high expression values. In network modeling of pathways, the PCI of each pathway is further normalized with its size *N*. This makes the PCI across all pathways follow approximately a normal distribution. PCI incorporates information of all available genes in the pathway. Subtle yet consistent gene expression modification will lead to a significant change in PCI. Furthermore, PCI captures the topological properties of the pathway, hub genes contribute more to PCI [13].

### Networking pathways and traits

**Figure 1:**
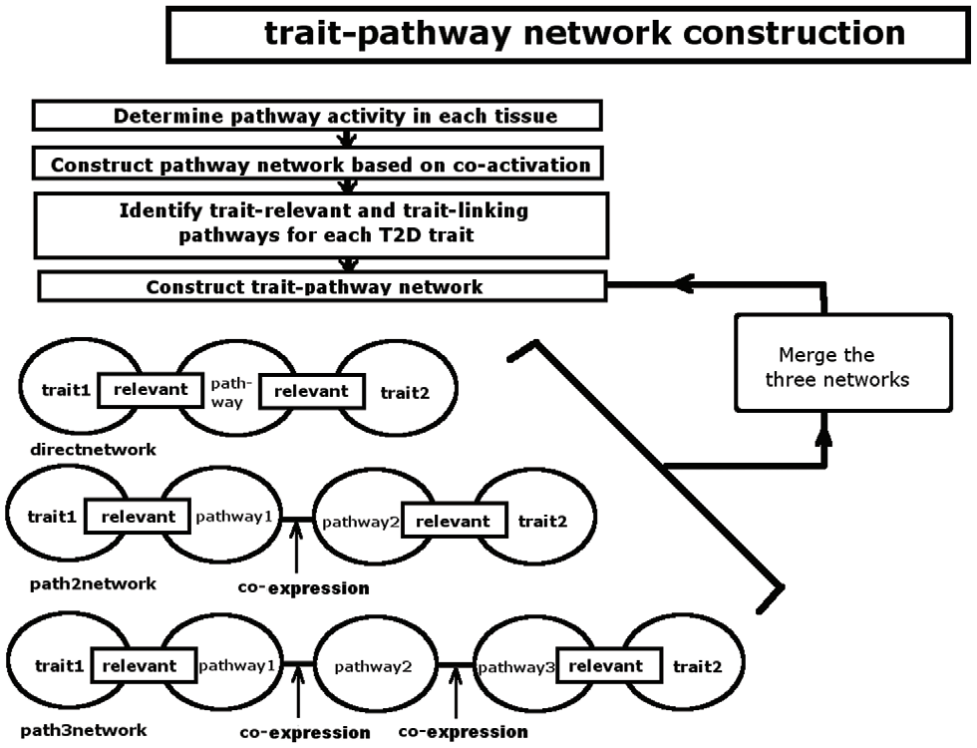
The pipeline of cross tissue trait-pathway network construction.

We propose a pipeline to construct the network of pathways and traits by integrating the multi-tissue gene expression, trait measurements, and pathways from KEGG (www.genome.jp/kegg/). The scheme of the pipeline is given in Figure 1.

First the PCI of each pathway in each tissue of each animal is determined. Next a pathway coordination network is constructed where each node represents a pathway in a certain tissue and hence has two attributes: {pathway, tissue}. An edge is added between two nodes if their pathway PCIs correlate and the pathways are curated to be linked by the KEGG. This includes the situations of two different pathways in the same tissue, two different pathways that each is from a different tissue, and the same pathway in two different tissues.

Thirdly, we construct the trait-pathway network. For each T2D trait, we define the relevant and the linking pathways. A relevant pathway is one whose PCI correlates significantly with the trait variation. A pathway is trait-linking if it satisfies one of the following three conditions (Figure 1): (1) is a relevant pathway simultaneously for 2 or more traits; (2) is relevant to one trait and interacts with a relevant pathway of another trait; (3) interacts simultaneously with relevant pathways of two different traits. These three situations are depicted at the bottom of Figure 1. We called them the Direct-network, Path2-network, and Path3-network, respectively. Merging them we obtain a complex trait-pathway network which links the T2D related traits and pathways in the six key tissues. The network was further refined by removing nodes of network degree 0 or 1 and those where the sum of distance to the two nearest traits is >6. This helps to focus the analysis on those nodes that are more interactive and more likely to be relevant to T2D traits.

Both the global networks of all animals and the networks specific to each of the eight groups of mice were constructed. In this study we used Spearman correlation coefficient to construct the pathway co-expression network. The threshold values were determined through permutation tests. Briefly, the expression data of different genes were randomly permuted 10,000 times, all pairwise pathway correlation coefficients were recalculated each time. Using the permutation results, the p-value for each correlations value is then determined. We chose p<0.005 as the threshold for significant correlation, this correspond to *r*>0.7 as the threshold for correlation when constructing networks in each of eight groups of mice, and *r*>0.4 when constructing shared network for all 40 mice. We have also tried other correlation indices, including the percentage bend correlation index [19], and found no significant difference in results obtained.

Fisher's exact test was used to evaluate enhancement in pathway interaction as compared to the KEGG annotation, and multiple testing was corrected using the false discovery rate (FDR) [20].

### Topological analysis and visualization of the trait-pathway network

A Java script based on the Cytoscape library was developed to analyze the topological properties of the trait-pathway network, including the network degrees, betweenness, and the distributions of linking pathways and “hub” pathways in different tissues. Edge betweenness, *i.e.* the fraction of shortest paths between all other nodes that pass through a given node in the network, were determined adopting Ulrik Brandes' algorithm [21]. The MCODE Cytoscape plug-in [22] was used to identify densely connected subregions of the network, namely, clusters of highly interactive pathways. These clusters are usually of core importance for the function of the whole network. Pajek [23] (http://pajek.imfm.si/) was used to visualize trait pathway network with partition of the nodes based on pathway category and tissues types.

## Results

### Associating tissue specific pathways and T2D quantitative traits

Using all animals, we identified a number of trait-relevant tissue-specific KEGG pathways: 186 for insulin, 32 for glucose, 19 for islet number, 34 for PAI1, 100 for resistin, 107 for TG, 209 for weight, and 9 for adiponectin. Table 1 lists the top tissue-specific pathways that are significantly correlated with the plasma insulin level. Many studies have suggested the relevance of these pathways to obesity and diabetes, such as Biosynthesis of unsaturated fatty acids, Fatty acid biosynthesis, Butanoate metabolism, and Oxidative phosphorylation [4]. The complete list of trait relevant pathways is available in Table S1.

**Table 1:** Top insulin-relevant tissue-specific KEGG pathways.

Tissue	Pathway
Liver	Biosynthesis of unsaturated fatty acids
Liver	Fatty acid biosynthesis
Islet	Phenylalanine metabolism
Liver	Benzoate degradation via CoA ligation
Liver	Glycerophospholipid metabolism
Islet	Biotin metabolism
Islet	Benzoate degradation via CoA ligation
Islet	Lysine biosynthesis
Adipose	Systemic lupus erythematosus
Islet	Glycosylphosphatidylinositol(GPI)-anchor biosynthesis
Islet	Bisphenol A degradation
Adipose	Aminosugars metabolism
Adipose	Gap junction
Liver	Butanoate metabolism
Liver	PPAR signaling pathway
Islet	Ribosome
Adipose	Non-small cell lung cancer
Liver	Pentose phosphate pathway
Liver	Glutathione metabolism
Adipose	Melanoma
Islet	Protein export
Liver	Geraniol degradation
Liver	Starch and sucrose metabolism
Adipose	Fc epsilon RI signaling pathway
Islet	Nucleotide sugars metabolism
Islet	Oxidative phosphorylation
Liver	Pyruvate metabolism
Liver	Valine, leucine and isoleucine degradation
Islet	D-Arginine and D-ornithine metabolism
Liver	Thiamine metabolism
Liver	Bile acid biosynthesis
Liver	Metabolism of xenobiotics by cytochrome P450
Adipose	Renal cell carcinoma
Adipose	SNARE interactions in vesicular transport
Adipose	Glioma

The co-expression of KEGG pathways were determined based on their linkage annotated in KEGG and the correlative variations in their PCI. We found that the pathways annotated to be linked by the KEGG database share high PCI correlation both intra and cross tissue. The correlation is significant even after adjusting for the number of shared genes between the different pathways (data not shown). The cross tissue pathway co-expression is likely brought about by the communication between different tissues and suggests the importance of characterizing such crosstalk during disease.

**Figure 2:**
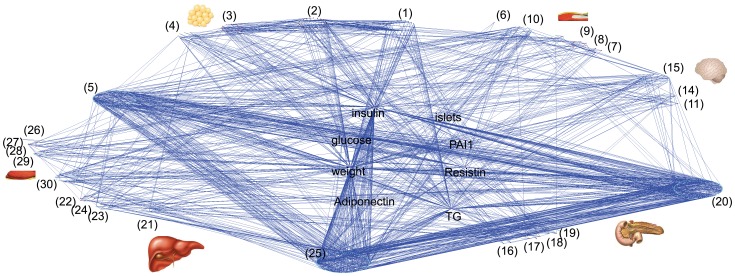
The trait-pathway network of T2D. The pathways are clustered based on tissue type and KEGG pathway category. The largest clusters are the metabolic pathways in adipose and liver.

Utilizing the trait-relevant and trait-linking pathways and the pathway co-expression network, we constructed the trait-pathway network, which is given in Figure 2. The association among the 8 T2D traits and communications between different tissues are depicted through the trait-pathway and the pathway-pathway links. In total there are 192 trait linking pathways in the Direct-network, 228 in Path2-network, and 352 in Path3-network. The final network contains 405 pathway/tissue nodes and 2099 interactions. Note that each node has two attributes: {pathway, tissue}. A SIF version of network was put into Cytoscape webcast and is available at http://zen.dom.uab.edu:8080/pathwaynetwork/cytoscape/, where one can view the details of the network with several built-in operations and examine each node and its neighbors. Most of the pathways are metabolism or inflammation related, which is consistent with the current understanding of T2D. To appreciate the general theme of this very complex figure, pathways that are in the same tissue and are from the same KEGG pathway categories were clustered together. The categories include Metabolism, Genetic information processing, Environment information processing, Cellular possesses (some pathways in this category were later taken out and expanded into a new category: Organismal systems), and Human diseases. The presence of pathway members from each category is further evaluated using Fisher's exact test. The results for all 30 clusters (5 KEGG categories×6 tissues) are given in Table 2. We found that in some tissues, members of certain pathway categories exhibit significantly enhanced presentation in the trait-pathway network. The top 3 clusters are Metabolism pathways in liver, islet and adipose tissue, indicating that they are the most active and interactive and, hence, the most relevant to disease traits. Four out of five pathway categories are active in the adipose tissue, which suggests a primary involvement of this tissue in obesity-induced diabetes.

**Table 2:** The pathway categories in the trait-pathway network.

Index^1^	KEGG pathway category	Tissue	Fisher p-value, Over-representation	Fisher p-value, under-representation
25	Metabolism	Liver	**2.91E-09**	1
20	Metabolism	Islet	**3.63E-06**	1
5	Metabolism	Adipose	**0.0019**	1
1	Human diseases	Adipose	**0.0038**	0.998
2	Cellular possesses	Adipose	**0.0038**	0.998
3	Environment information processing	Adipose	**0.040**	0.984
4	Genetic information processing	Adipose	0.090	0.961
8	Environment information processing	Gastrocnemius	0.388	0.772
14	Genetic information processing	Hypothalamus	0.474	0.710
18	Genetic information processing	Islet	0.474	0.710
9	Genetic information processing	Gastrocnemius	0.580	0.623
23	Genetic information processing	Liver	0.580	0.623
29	Genetic information processing	Soleus	0.580	0.623
28	Environment information processing	Soleus	0.904	0.237
17	Cellular possesses	Islet	0.920	0.164
22	Cellular possesses	Liver	0.920	0.164
15	Metabolism	Hypothalamus	0.952	0.0766
16	Human diseases	Islet	0.961	0.0951
7	Cellular possesses	Gastrocnemius	0.984	**0.0481**
11	Human diseases	Hypothalamus	0.984	**0.0481**
10	Metabolism	Gastrocnemius	0.986	**0.0253**
19	Environment information processing	Islet	0.993	**0.0443**
24	Environment information processing	Liver	0.993	**0.0443**
21	Human diseases	Liver	0.994	**0.0202**
26	Human diseases	Soleus	0.994	**0.0203**
30	Metabolism	Soleus	0.999	**0.0033**
6	Human diseases	Gastrocnemius	1	**0.0015**
27	Cellular possesses	Soleus	1	**0.0015**
13	Environment information processing	Hypothalamus	1	**0.0101**
12	Cellular possesses	Hypothalamus	1	**0.000201**

1 : Cluster index in Figure 1.

It was previously observed that known disease genes generally exhibit tissue specific activity, with expression levels altered in the tissues where specific gene defects cause pathology [8], [24]. Therefore we also examined the distribution of the trait-linking pathways in the six T2D relevant tissues. The results are listed in Table 3. Evidently, the pathways are not equally distributed in the six tissues. Adipose tissue contains the largest number of trait-linking pathways for all traits, consistent with the fact that obesity is the primary cause of T2D in this animal model. In addition, the eight traits have a wide range of linking pathways. Weight possesses the most, again maybe an indication of the central role of obesity in T2D. Insulin level has the second largest number, which fits well with the fact that impaired compensatory insulin secretion under obesity is also a causal factor in T2D.

**Table 3:** Distribution of trait-linking pathways in six tissues.

Trait	Total # of pathways	Adipose	Gastrocnemius	Hypothalamus	Islet	Liver	Soleus
TG	106	40	6	5	25	25	5
glucose	31	5	2	3	11	9	1
Adiponectin	8	1	1	0	2	4	0
Resistin	53	22	7	1	8	9	6
PAI1	23	10	1	2	3	5	2
Insulin	177	51	11	5	43	61	6
weight	185	63	14	14	32	47	15
islets	16	4	2	0	6	4	0
Total each tissue	599 (in all tissues)	196	44	30	130	164	35

### Topological properties of the trait-pathway network

**Figure 3:**
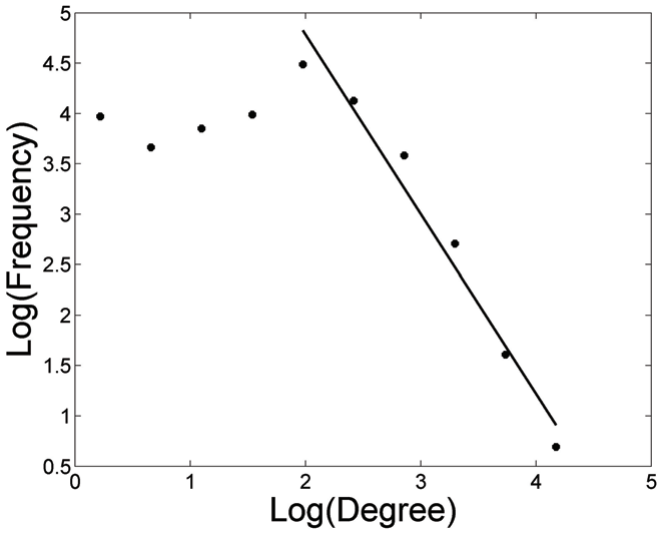
The degree distribution of the trait-pathway network. Axes are in natural base log scale. The network degree (number of interactions with other pathways) of pathways was log transformed and allocated into 10 evenly spaced bins. Plotted are the frequency counts in each bin against the bin center value.

We first investigated the global topological properties of the trait-pathway networks in all six tissues. The degree distribution of the network nodes is given in Figure 3. Interestingly it exhibits a mixed behavior. While for nodes with degrees higher than 8 there is clearly a power-law dependence, the frequencies for nodes with degree below 8 are flat. This is different from the scale-free networks typically observed with gene or protein networks. The leveling off at the low degree end suggests that there are not as many nodes with very low number of interactions as in a scale free network. This is reasonable for a KEGG pathway network, as these pathways are all of important functions and their interactions are well annotated.

The power-law dependence at degrees >8 is indicative of a small proportion of pathways serving as hub nodes that connect to a large number of other pathways. In the study of gene or protein networks, highly connected nodes are often considered important to network function. For instance, analysis of the yeast protein-protein interaction network revealed that the highly connected genes are more likely to be essential for survival [25]. It is tempting to hypothesize here that the highly-connected pathways in the trait-pathway network are the most relevant to the obesity induced T2D. Table 4 lists the top 20 highly connected tissue-specific pathways. The most connected pathway is mmu00010, Glycolysis/Gluconeogenesis in the liver, which interacts with 82 other pathways. Its degrees in adipose tissue and islet are also high, 40 and 30, respectively. T2D is a complex disorder where diminished insulin secretion and impaired insulin action together lead to chronic hyperglycemia. The glucose metabolic pathways play an important role in the pathogenesis of the disease. For instance, glycolysis is a critical step in the signaling pathway of glucose stimulated insulin secretion in islets [26]. Abnormalities in them have been observed in diabetic subjects [27].

**Table 4:** Top 20 tissue-specific pathways with highest degrees.

Tissue	Pathway	Description	Degree
Liver	mmu00010	Glycolysis/Gluconeogenesis	82
Liver	mmu00020	Citrate cycle (TCA cycle)	55
Liver	mmu00252	Alanine and aspartate metabolism	46
Liver	mmu00620	Pyruvate metabolism	45
Adipose	mmu00010	Glycolysis/Gluconeogenesis	40
Liver	mmu00260	Glycine, serine and threonine metabolism	37
Islet	mmu00620	Pyruvate metabolism	37
Liver	mmu00030	Pentose phosphate pathway	34
Islet	mmu00230	Purine metabolism	33
Islet	mmu00630	Glyoxylate and dicarboxylate metabolism	32
Islet	mmu00010	Glycolysis/Gluconeogenesis	30
Liver	mmu00630	Glyoxylate and dicarboxylate metabolism	29
Liver	mmu00280	Valine, leucine and isoleucine degradation	26
Hypothalamus	mmu00040	Pentose and glucuronate interconversions	26
Adipose	mmu00400	Phenylalanine, tyrosine and tryptophan biosynthesis	26
Adipose	mmu00230	Purine metabolism	25
Adipose	mmu04010	MAPK signaling pathway	25
Liver	mmu00040	Pentose and glucuronate interconversions	24
Liver	mmu00251	Glutamate metabolism	24
Liver	mmu00220	Urea cycle and metabolism of amino groups	24

The citric acid cycle (TCA) is series of chemical reactions used by all aerobic organisms to generate energy. It breaks down pyruvates from glycolysis (and other pathways), and generates ATP by oxidative phosphorylation. The importance of the TCA cycle and the pyruvate metabolism pathway in obesity and T2D has been demonstrated [28], [29]. TCA cycle and/or oxidative phosphorylation flux is known to be reduced in diabetes compared to healthy controls [30], [31]. Additionally some evidence suggests that the reduced TCA flux may be of primary origin and may control several major diabetic phenotypes including the increased basal glucose uptake, increased basal glucose oxidation, and reduced complete lipid oxidation [32]. In addition pyruvate is metabolized in pancreatic islets, though the extent and mechanism of this metabolism remains unclear.

Edge betweenness, which measures the fraction of shortest paths going through a node, is an important centrality index [33]. It captures the influence that an individual node has in the spread of information within the network [33]. In yeast it was observed that proteins with high betweenness are more likely to be essential [34]. The top pathways with highest betweenness in our trait-pathway network are listed in Table 5. Insulin signaling pathway in gastrocnemius ranks second among all pathways. It interacts with several other pathways important in glucose homeostasis [27]. Insulin increases glucose uptake and metabolism in skeletal muscle by signal transduction via protein phosphorylation cascades. Some downstream intermediates in the insulin signaling pathways govern glucose homeostasis and can lead to skeletal muscle insulin resistance in T2D [35]. Insulin action on signal transduction is impaired in skeletal muscle from T2D subjects, and the dysfunction is key to T2D development [36]. The MAPK signaling, together with T cell receptor signaling pathway, constitute another pathologically meaningful component relevant to obesity induced diabetes: the inflammation in adipose tissue [37]–[39].

**Table 5:** Top 20 tissue-specific pathways with highest betweenness.

Tissue	Pathway	Description	Score
Liver	mmu00010	Glycolysis/Gluconeogenesis	34451
Gastrocnemius	mmu04910	Insulin signaling pathway	20871
Adipose	mmu04120	Ubiquitin mediated proteolysis	16827
Adipose	mmu04010	MAPK signaling pathway	12459
Islet	mmu00350	Tyrosine metabolism	12147
Adipose	mmu00010	Glycolysis/Gluconeogenesis	9971
Liver	mmu00020	Citrate cycle (TCA cycle)	9704
Gastrocnemius	mmu04150	mTOR signaling pathway	8510
Liver	mmu00260	Glycine, serine and threonine metabolism	8480
Adipose	mmu04660	T cell receptor signaling pathway	8294
Liver	mmu00564	Glycerophospholipid metabolism	7873
Gastrocnemius	mmu04010	MAPK signaling pathway	7642.
Liver	mmu00620	Pyruvate metabolism	7444
Liver	mmu00350	Tyrosine metabolism	7050
Islet	mmu00010	Glycolysis/Gluconeogenesis	6789
Adipose	mmu00051	Fructose and mannose metabolism	6564
Liver	mmu00051	Fructose and mannose metabolism	6330
Islet	mmu00563	Glycosylphosphatidylinositol (GPI)-anchor biosynthesis	6203
Islet	mmu00230	Purine metabolism	5813
Islet	mmu05020	Parkinson's disease	5681

**Figure 4:**
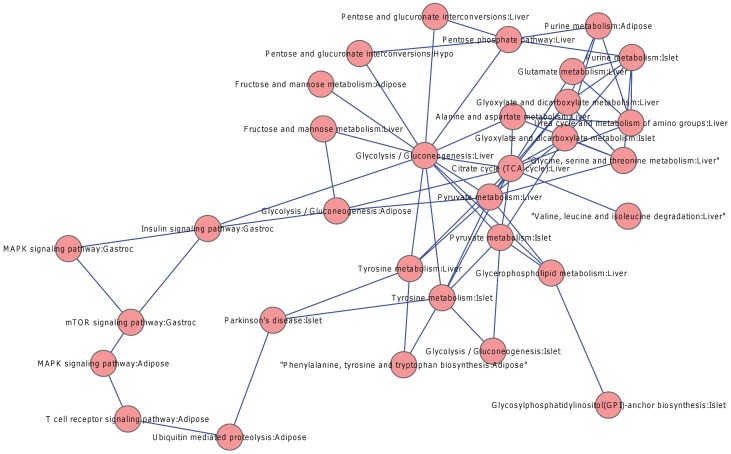
Core of the T2D trait-pathway network. Nodes are the top high-degree and high-betweenness pathways from Tables 4 and 5.

In scale-free networks, high degree nodes tend to have high betweenness. In our trait-pathway network, most of the pathways with high betweenness also have high degree (compare Table 5 with Table 4, 8 of the 20 elements are shared). Figure 4 depicts the subnetwork of the top high-degree and high-betweenness pathways listed in Tables 4 and 5. We hypothesize that together they constitute a signature pathway network core of T2D, which provides a picture of the primary molecular basis of T2D pathogenesis.

### Densely connected subregions of the trait-pathway network

**Figure 5:**
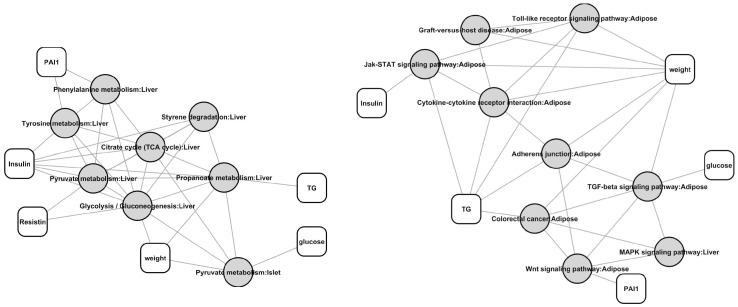
Pathway composition of the top 2 densely connected subregions of the whole trait-pathway network.

Using MCODE [22] we found that the trait-pathway network contains a number of densely connected subregions. The top 2 are displayed in Figure 5. They contain 8 nodes and 20 edges, and 9 nodes and 14 edges, respectively.

7 out of the 8 pathways in the first cluster are in liver tissue. As discussed in the previous sections, glycolysis, TCA cycle, and pyruvate metabolism pathways are well known to be T2D related. Protein tyrosine phosphatase 1B (PTP1B) has been found to be a major regulator of body fat stores, energy balance, and insulin sensitivity in vivo. Increased expression of PTP1B is associated with insulin resistance in rodents and humans whereas deletion of PTP1B leads to leanness and insulin sensitivity in rodents [40]. Propionate can lower blood glucose and alters lipid metabolism in healthy subjects [41].

8 out of the 9 pathways in the second cluster are in adipose tissue. MAPKs are intracellular signaling pathways that play a pivotal role in many essential cellular processes such as proliferation and differentiation, specifically in adipocyte differentiation and obesity [42]. Transforming growth factor-beta/Smad3 signaling regulates insulin gene transcription and pancreatic islet β-cell function [43]. Impaired insulin signaling and β-cell function is critical to obesity induced diabetes. Wnt signaling plays an important role in intestinal tumorigenesis and has been linked to susceptibility to T2D [44]. Carriers of variants of the transcription factor 7-like 2 gene, an important component of the Wnt pathway, are at increased risk for developing T2D. The modulation of proglucagon expression by Wnt activity may partially explain the link between Wnt signaling and diabetes. Insulin resistance-inducing cytokines differentially regulates SOCS mRNA expression via growth factor- and Jak/Stat-signaling pathways in 3T3-L1 adipocytes [45].

### Context specific network reveals the importance of the OXPHOS and the T cell receptor signaling pathway in obesity-induced diabetes

The trait-pathway network presented in Figure 2 is constructed using both existing information of pathway links annotated in KEGG and the expression data specific to the animals of an obesity-induced diabetes model. The pathway interactions annotated by KEGG are not specific to obesity or diabetes. They are generic summaries of the most common denominator from many instances and likely represent the most conserved parts of the interaction network. On the other hand, the expression data offers insight into the activities occurring in the animal model and hence information more specific to the disease under investigation. Using Fisher's exact test we identified pathways where the expression data brought in significantly more interaction linkages. The top 15 pathways are given in table 6. Most of them are in liver and adipose, suggesting that pathway interactions are most active in these tissues during the obesity development.

**Table 6:** Top pathways with highest degree after adjusted by the number of curated interactions.

Tissue	Pathway	Name	Network degree	FDR
Liver	mmu00010	Glycolysis/Gluconeogenesis	82	8.94E-05
Liver	mmu00280	Valine, leucine and isoleucine degradation	26	0.0063
Adipose	mmu00900	Terpenoid biosynthesis	13	0.0228
Liver	mmu00030	Pentose phosphate pathway	34	0.0358
Gastrocnemius	mmu00052	Galactose metabolism	18	0.0753
Gastrocnemius	mmu00562	Inositol phosphate metabolism	12	0.0835
Adipose	mmu00532	Chondroitin sulfate biosynthesis	15	0.0898
Liver	mmu00310	Lysine degradation	15	0.0898
Liver	mmu00053	Ascorbate and aldarate metabolism	23	0.0960
Liver	mmu00252	Alanine and aspartate metabolism	46	0.0982
Liver	mmu00020	Citrate cycle (TCA cycle)	55	0.226
Liver	mmu00740	Riboflavin metabolism	14	0.264
Adipose	mmu00603	Glycosphingolipid biosynthesis - globoseries	11	0.287
Adipose	mmu00562	Inositol phosphate metabolism	11	0.287
Islet	mmu00604	Glycosphingolipid biosynthesis - ganglioseries	11	0.287

Furthermore, we constructed a group-specific network for each of the 8 animal groups in addition to the global network for all animals. The networks in Cytoscape, viewable SIF format files, are given in Figure S1, which is also available at http://zen.dom.uab.edu:8080/pathwaynetwork/cytoscape. The basic network characteristics include the total number of nodes and edges are summarized in Table S2. Table 7 lists the number of trait relevant pathways in each of the eight networks. Interestingly, while in general the number increases moderately from 4 wk to 10 wk for most animals, the B6 ob 10 wk animals showed a significant drop, from 258.4 in average to 102.8. These are obese animals that do not develop diabetes. The pathways with enhanced interactions compared to KEGG annotations are given in the Table S3. The most interesting patterns are again observed in the B6-obese-10 wk group, which does not develop diabetes; and in the BTBR-obese-10 wk group (Table 8), which is diabetic. Together they suggest that in both groups the pathways relevant to glucose metabolism and oxidative stress are interacting actively with other pathways in these animals. In the BTBR-obese-10 wk group additional pathways relevant to inflammations are also active, suggesting that they may be the key differentiating factor that determines why some obese animals develop diabetes and some do not. It is known that obesity is associated with a state of chronic, low-grade inflammation [37]. Inflammatory and stress responses mediate insulin resistance. Obesity-induced inflammation and the signaling pathways at the intersection of metabolism and inflammation contribute to diabetes.

**Table 7:** Number of trait-relevant pathways in each of the eight animal groups.

Trait	B6-ob 4 wk	B6-ob 10 wk	B6-lean 4 wk	B6-lean 10 wk	BtBr-ob 4 wk	BtBr-ob 10 wk	BtBr-lean 4 wk	BtBr-lean 10 wk
Adiponectin	233	0	0	0	145	277	0	152
glucose	295	147	189	211	270	229	142	0
Insulin	346	155	101	193	239	151	187	207
islets	170	0	0	0	67	159	0	0
Leptin	257	179	167	196	259	197	121	243
Resistin	250	0	0	0	95	178	0	0
TG	323	165	139	211	231	228	175	186
weight	193	176	183	288	198	265	211	197
**Mean**	**258.4**	**102.8**	**97.4**	**137.4**	**188.0**	**210.5**	**104.5**	**123.1**

**Table 8:** Pathways with the most significant interactions in the BTBR obese 10 week mice.

Tissue	Pathway	Name	Network degree	p-value	FDR
Hypothalamus	mmu00190	Oxidative phosphorylation	27	8.5E-07	0.00063
Islet	mmu04660	T cell receptor signaling pathway	58	1.1E-05	0.0080
Soleus	mmu05332	Graft-versus-host disease	35	0.00045	0.331
Adipose	mmu05332	Graft-versus-host disease	35	0.00045	0.331
Adipose	mmu05320	Autoimmune thyroid disease	31	0.00065	0.477
Gastrocnemius	mmu00190	Oxidative phosphorylation	20	0.00072	0.532
Adipose	mmu04510	Focal adhesion	20	0.00072	0.532
Adipose	mmu04940	Type I diabetes mellitus	16	0.00076	0.556
Hypothalamus	mmu05012	Parkinsons disease	30	0.0012	0.911
Adipose	mmu05330	Allograft rejection	30	0.0012	0.912
Adipose	mmu05012	Parkinsons disease	30	0.0012	0.912
Gastrocnemius	mmu00760	Nicotinate and nicotinamide metabolism	23	0.0013	0.934
Gastrocnemius	mmu04514	Cell adhesion molecules (CAMs)	43	0.0013	0.978

**Figure 6:**
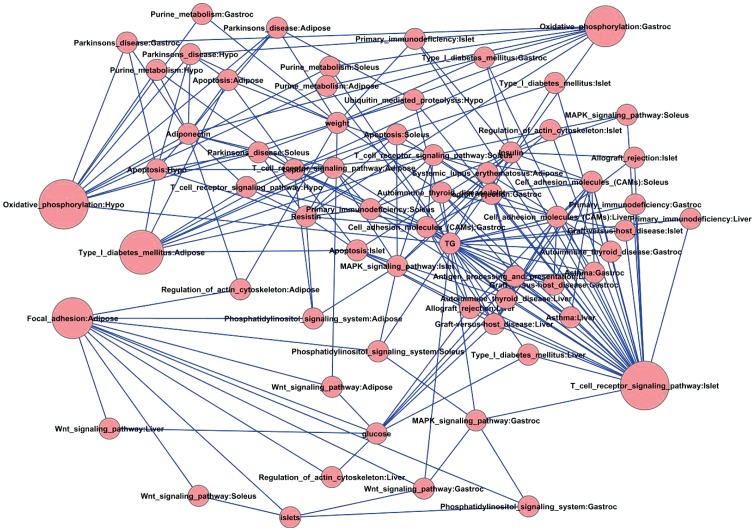
Trait-pathway network for the BTBR obese 10 week mice. Nodes are the quantitative traits and pathways with FDR<0.3 and their first-degree neighbors. Node sizes are defined by the enhancement significance of node degree (5^th^ column, p-value, of Table 8).

In more detail, the top overrepresented pathways and their first neighbors in the diabetic BTBR-obese-10 wk group are displayed in Figure 6. The top pathways include oxidative phosphorylation in hypothalamus and T cell receptor signaling in islet. Recently, increasing experimental and clinical evidence suggests that hypothalamic dysregulation may be one of the underlying mechanisms of abnormal glucose metabolism and may underlie at least some portion of T2D or insulin resistance in humans [46]. Hypothalamus shares with pancreas several commonly expressed molecules that are critical in glucose sensing and inhibition of insulin action on hepatic gluconeogenesis. Mouse model studies revealed that obesity induced hypothalamic resistance to insulin may be involved in pathogenesis of peripheral insulin resistance [47]; however, the exact role and mechanism of hypothalamus involvement in T2D is still not clear.

Microarray studies have shown that genes involved in oxidative phosphorylation (OXPHOS) exhibit reduced expression levels in the skeletal muscle of T2D and prediabetic subjects. These changes may be mediated by the peroxisome proliferator–activated receptor γ coactivator-1 (PGC1) pathway. Decreased expression of PGC1α- and PGC1β-responsive OXPHOS genes in muscle, and of genes involved in oxidative phosphorylation in pancreatic islets, were observed in T2D patients [10], [31]. The importance of OXPHOS genes are also supported by genetics study [48]. Our analysis results for the first time revealed that the OXPHOS pathway is actively interacting with other pathways in hypothalamus in diabetic animals, suggesting that oxidative stress in hypothalamus may be the underlying mechanism in obesity induced diabetes.

One critical contributing factor to obesity induced diabetes is the inadequate insulin secretion resulting from β-cell death [49]. The death occurs as a consequence of increased circulating glucose, saturated fatty acids, adipocyte secreted factors, and chronic activation of the innate immune system. In both type 1 and type 2 diabetes intra-islet inflammatory mediators seem to trigger a final common pathway leading to β-cell apoptosis. Anti-inflammatory therapeutic approaches designed to block β-cell apoptosis could be a significant new development [50]. The active role identified here of the T cell receptor signaling pathway can help to narrow down the potential therapeutic targets.

## Discussion

In this study, we proposed an approach to build tissue-specific, disease trait-pathway networks through associating co-activated pathways, and trait-relevant and trait-linking pathways. Our strategy goes beyond single gene based analysis. It utilizes the PCI to capture the overall activity of each pathway under given experimental conditions, which incorporates contributions from both the individual genes and how they are geometrically situated in the network. The PCI measure is then used to infer interactions between pathways and between T2D traits and pathways. To our knowledge, this is the first of its kind that delineates the disease traits at the genetic pathway level.

The new approach revealed a number of findings compared to original analysis by the authors that generated the dataset [7]. It identified a set of pathways that are responsible for the association among the main T2D traits. The cross tissue pathway networks highlighted communication among the insulin releasing and insulin target tissues. Topological analysis of the network revealed that many pathways that are of topological importance to the network, *i.e.*, those with high degree and high betweenness, are closely involved in glucose metabolism and insulin. A core of pathway clusters was identified that may provide a relatively complete view of the key pathways and their interactions that potentially mediate disease pathophysiology for T2D. In a group-specific analysis, we found a difference in active pathways between the obese animals that developed diabetes and those that did not. While those involved in glucose metabolism and oxidative stress are interactive in both groups, those involved in inflammation exhibit enhanced interaction with other pathways only in the obese animals that develop diabetes. We believe that such multi-scale (genes, pathways, and tissues) systems analysis will provide valuable insight into disease etiology and is essential to better understand the pathophysiology and the pathogenesis of a complex disease like T2D.

Obesity induced diabetes is a complex issue; multiple pathways in many tissues are involved. The bioinformatics study presented in this work offers a glimpse of the underlying genetic architecture. There are still many questions to be answered. For instance, in table 7, we see that, in general, the number of trait-relevant pathways increases with age (which is itself a risk factor for obesity and diabetes). The obese animals that do not develop diabetes show a marked reduction in the number of trait-relevant pathways. It is very intriguing then whether the pathways that showed differential trait-relevance contain key information as to why some obese individuals develop diabetes while some don't. These deserve further investigation in future.

In this study we only analyzed data from two mouse strains. It will be interesting to compare the trait-pathway networks across different models of T2D, which would be valuable to understanding the disease etiology and how well each model represents the human disease. This approach can be generalized from the analysis of multiple traits of one disease to the interrelations among a set of different diseases. Then diseases can be linked through their relevant and linking pathways. The problem of associating multiple diseases is subsequently converted to the problem of network comparison. Finally, it would also be of interest to study trait-pathway networks in general across different species. The results will shed light to the functional evolution of related pathways and pathway interactions.

## Supporting Information

Table S1 **The tissue-specific pathways relevant to each trait.**
(XLS)

Table S2 **Network measures, including the total number of nodes and edges, of the trait relevant pathway network in each of weight animal groups.**
(XLSX)

Table S3 **Pathways with enhanced interactions in each of the eight animal groups.**
(XLSX)

Figure S1 **Trait-pathway networks in 8 groups in Cytoscape viewable SIF format. (A) B6 lean 4 week. (B) B6 lean10 week. (C) B6 obese 4 week. (D) B6 obese 10 week. (E) BTBR lean 4 week. (F) BTBR lean10 week. (G) BBR obese 4 week. (G) BTBR obese 10 week.**
(ZIP)
